# Operative Management of Perforated Jejunal Diverticulitis

**DOI:** 10.7759/cureus.21330

**Published:** 2022-01-17

**Authors:** Deena Abdelhalim, Thomas Kania, Audrey Heldreth, Nicholas Champion, Indraneil Mukherjee

**Affiliations:** 1 Surgery, Staten Island University Hospital, Staten Island, USA

**Keywords:** small bowel resection, small bowel diverticulitis, perforation, jejunal, diverticulitis

## Abstract

Small jejunal diverticulitis is very rare, presenting in 0.06% to 1.3% of the population. Many patients remain asymptomatic or have nonspecific symptoms such as malabsorption and abdominal pain, making diagnosis complicated. Up to 6% of patients present with acute perforation. Here, we present such a case involving a 69-year-old female who presented with altered mental status due to sepsis and generalized peritonitis from a perforated jejunal diverticulum that was successfully managed with definitive surgery. We highlight the importance of maintaining a broad differential, early resuscitation, and prompt surgical management in complicated jejunal diverticulitis. Although adjunctive studies such as computed tomography may be helpful in stable patients, definitive surgery was both diagnostic and therapeutic in this case.

## Introduction

Jejunal diverticulosis is characterized by multiple herniations of the mucosa and submucosa through the muscular layer of the bowel. These diverticula are thought to occur through weaknesses in the muscular layer at sites where the vasa recta enter the muscularis propria [1]. As described, the pathophysiology of jejunal diverticulitis is that of pulsion-type false diverticula because the diverticula themselves do not include the muscular layer of the bowel. This is in contrast to the true diverticula of the jejunum which have also been described; these are usually singular and are often identified as Meckel’s diverticula [2].

Small intestinal diverticula were first described by Sommering and Baille in 1794 and are very rare, with an incidence of 0.06% to 1.3%. The incidence is higher in the sixth to eighth decades of life and in men [3,4]. Duodenal diverticula are the most common of the small intestinal diverticula, accounting for approximately 79% of cases, followed by jejunal and ileal [5]. Many cases of small bowel diverticula remain asymptomatic or present with nonspecific symptoms such as malabsorption, abdominal pain, vomiting, and nausea, making diagnosis difficult. Only approximately 29% of patients develop signs and symptoms [6]. Although their pathogenesis is unclear, they have been proposed to arise from intestinal dyskinesia, abnormal peristalsis, and high intraluminal pressure [4].

Small intestinal diverticula are typically diagnosed incidentally on imaging or intraoperatively because symptoms are usually vague and can mimic many other disease processes. These include abdominal pain, pseudo-obstruction, and malabsorptive complications [7].

Overall, 10-30% of patients present with acute complications including perforation, obstruction, adhesions, fistulae, peritonitis, and lower gastrointestinal bleeding [8]. Perforation of jejunal diverticula is a severe complication that occurs in approximately 2-6% of cases [4]. In this setting, expeditious operative management is key. One must consider a broad differential diagnosis including jejunal or ileal diverticulitis and be prepared to perform a small bowel resection if necessary. Here, we describe the operative management of such a case.

## Case presentation

A 69-year-old woman presented to our institution complaining of a six-day history of abdominal pain and nausea. The patient had an extensive surgical history including diverticulitis, a Hartmann procedure, subsequent colostomy reversal, cholecystectomy, hysterectomy, and tubal ligation.

At the time of presentation, the patient described associated dysphagia and poor tolerance of her diet, along with a progressive worsening of the pain. She also described fevers up to 101°F and more than 10 watery bowel movements per day for the two days prior to her presentation.

In the emergency department, she was hemodynamically unstable with a blood pressure of 75/36 mmHg, heart rate of 94 beats per minute, temperature of 98.1°F, and oxygen saturation of 95% on room air. The patient was in clear discomfort but remained alert and oriented. Abdominal examination was remarkable for diffuse peritonitis with slightly more tenderness on her left side.

Intravenous fluid resuscitation was initiated with lactated Ringer’s solution administered as two 1 L boluses considering her hypotension and recent diarrhea. Laboratory studies showed an elevated white blood cell count of 26,000 cells/µL with a left-shift, a hemoglobin of 8.8 g/dL, hematocrit of 26.9%, sodium of 125 mmol/L, blood urea nitrogen of 77 mg/dL, creatinine of 6.3 mg/dL, and lactate of 1.1 mmol/L. Based on these results, her fluid resuscitation was transitioned to 0.9% normal saline which was administered as multiple additional boluses. Output data revealed oliguria, which did respond to fluid resuscitation.

**Figure 1:**
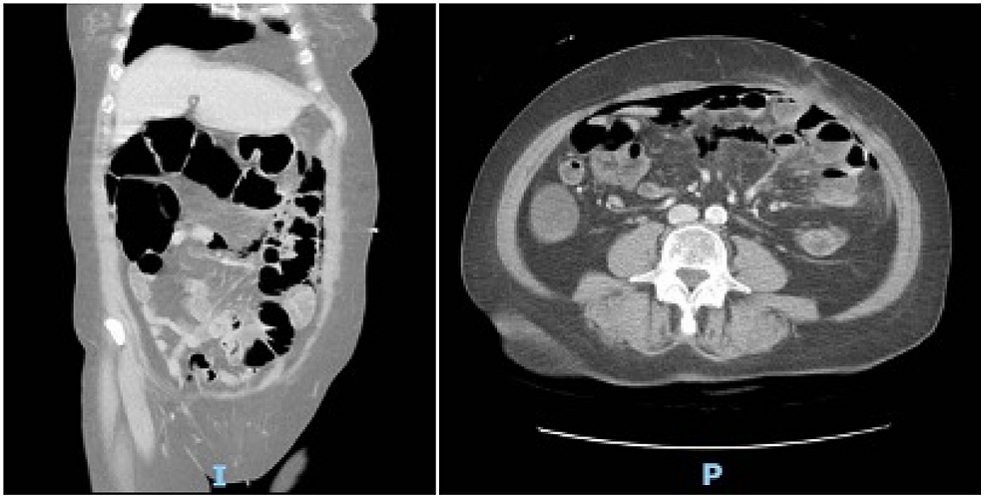
Representative coronal and axial sections demonstrating free intraperitoneal air located largely in the upper abdomen with concomitant inflammatory fat stranding in the left upper quadrant.

The patient’s hypotension also transiently responded to fluid resuscitation, and therefore, a computed tomography (CT) scan of the abdomen was performed with intravenous contrast. CT demonstrated moderate pneumoperitoneum and nonspecific inflammatory fat stranding in the left upper quadrant consistent with bowel perforation. No small bowel dilation, evidence of obstruction, or sigmoid diverticulitis or diverticulosis were appreciated (Figure 1).

Based on the CT findings, the surgical team suspected an upper gastrointestinal source of perforation. However, the patient’s blood pressure fell once again to 80/30 mmHg despite ongoing resuscitation with normal saline. Therefore, operative intervention was indicated and further diagnostic testing was not pursued. The patient’s clinical situation and the risks and benefits of surgery were discussed with the patient and her family and informed consent was obtained for surgery. A right internal jugular central venous catheter and left radial arterial line were placed and norepinephrine infusion was initiated. A Foley catheter was also inserted.

**Figure 2:**
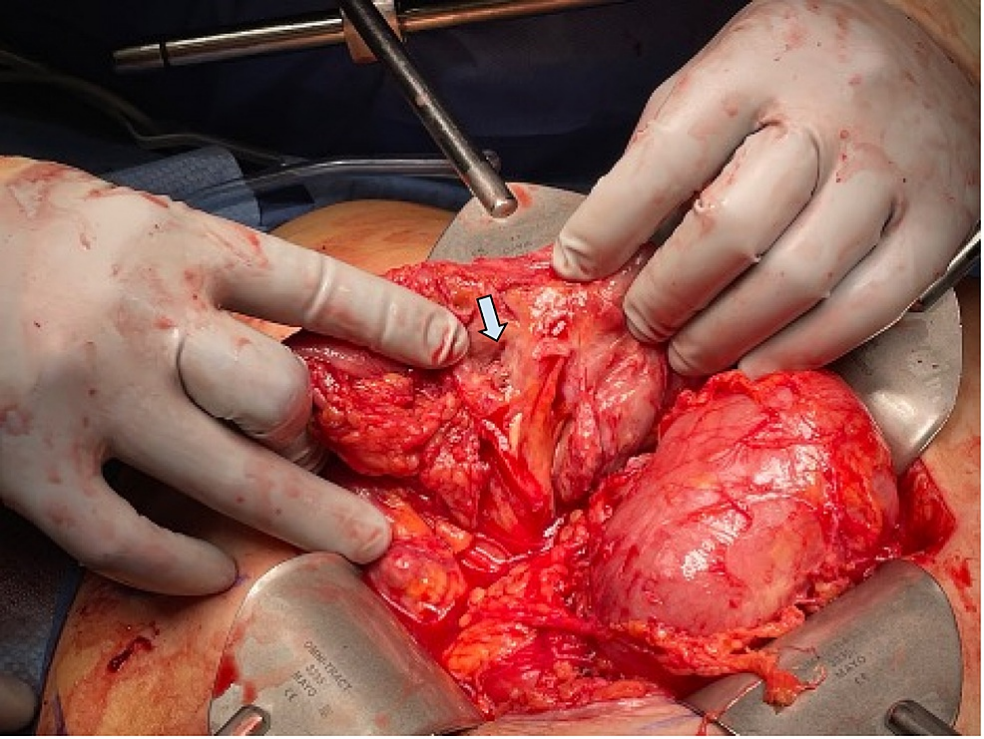
Site of the perforated jejunal diverticulum.

**Figure 3:**
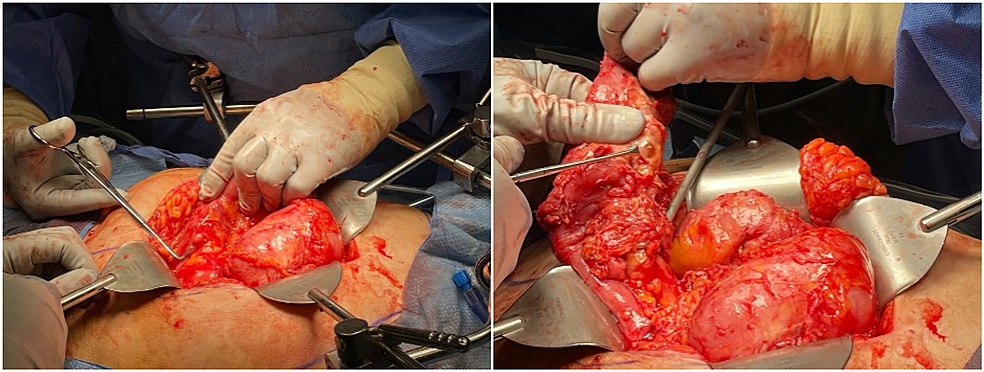
Additional nonperforated jejunal diverticulum, as indicated by the instrument tip.

Diagnostic laparoscopy revealed dense intra-abdominal adhesions. The procedure was quickly transitioned to an open approach due to ongoing hemodynamic instability. After extensive lysis of adhesions attributed to her previous surgeries, a perforated jejunal diverticulum was discovered with a 3 cm perijejunal abscess which was draining pus (Figure 2). This was opened and drained completely. Additional nonperforated diverticula were found throughout the affected segment (Figure 3). The remainder of the bowel was inspected, and the ileum and colon were unaffected.

Overall, 18 cm of jejunum was resected beginning at approximately 10-20 cm distal to the ligament of Treitz. A stapled anastomosis was performed. The procedure was completed without any complications, but the patient remained on norepinephrine infusion postoperatively.

She had a long but relatively uncomplicated postoperative course. She was extubated on postoperative day (POD) one. Her hypotension resolved and pressors were weaned by POD two. She was also managed expectantly for mild demand cardiac ischemia and pre-renal acute kidney injury. She experienced Intensive Care Unit (ICU) delirium and underwent a neurology evaluation as well as CT and magnetic resonance imaging of the head which revealed no focal deficits, evidence of ischemia, hematoma, or mass effect. She was started on total parenteral nutrition and was gradually transitioned to oral feeds. She was discharged from the ICU on POD four and was discharged home on POD 14.

The final pathology of the small bowel showed segments of diverticular disease with perforated diverticulum. There was significant inflammation in the bowel wall with extensive acute peritonitis, focal abscess formation, granulation tissue, and serosal adhesions.

## Discussion

Given the nonspecific symptoms of jejunal diverticulitis, the diagnosis is often unclear on initial presentation; therefore, it is important to maintain a broad differential diagnosis. If perforation has occurred and the patient is hemodynamically unstable, prompt resuscitation and surgical management are indicated. In our case, with an unclear etiology, ongoing resuscitation, and fluid responsiveness, imaging was obtained for further clarity.

Evaluation of such cases begins with a thorough history and physical examination. History should include risk factors for perforation such as recent endoscopy, surgery, history of cancer, inflammatory bowel or diverticular disease, and foreign body ingestion or insertion. The physical examination is crucial for identifying localized upper abdominal tenderness or, in our case, diffuse peritonitis.

Diagnostic CT scan is the gold standard for identification of diverticulitis, classically described as out-pouching lesions with thickened bowel walls [9]. In the absence of colonic diverticulitis, small bowel diverticulitis should be considered in cases of a perforated viscus. Other sources of perforation may include gastric and duodenal ulcers, iatrogenic injury, foreign bodies, and others. In our case, CT localized the pneumoperitoneum to the upper abdomen anteriorly, with inflammatory stranding of the mesenteric fat adjacent to the small bowel but no sign of obstruction. The location of such CT findings can provide clues regarding the site of perforation. This shifted our differential in favor of a small bowel or gastric source but ultimately did not alter the management in this patient with an acute abdomen.

Operative management was clearly indicated in this case involving a patient with peritonitis who only transiently responded to fluid resuscitation. When summarized as such, the preoperative decision-making is obvious, highlighting the continued importance of basic surgical principles in the modern era of ubiquitous CT scans. Once in surgery, such principles continue to guide the surgeon; diagnostic laparoscopy did not easily identify the source of perforation and was quickly transitioned to exploratory laparotomy. In retrospect, proceeding directly to exploratory laparotomy would have been preferable for this quasi-stable patient. A thorough examination of all of the bowel was also performed and is crucial in any case where the diagnosis or site of perforation is unclear. This allows one to identify any concurrent, otherwise potentially missed, disease [10].

Unlike this case, uncomplicated colonic and small intestinal diverticulitis can be managed nonoperatively with intravenous fluids, bowel rest, and antibiotics [8,11]. Perforated small bowel diverticulitis often requires surgical intervention. The mortality rate of jejunal diverticula ranges from 0% to 5%, but this risk increases to 40% in cases of perforation [11].

Although jejunal diverticulitis is rare, it is important to include it in the differential diagnosis of patients with abdominal pain and no colonic diverticulitis. Early diagnosis is key to prompt treatment to improve patient outcomes.

## Conclusions

Small bowel diverticulitis is an uncommon diagnosis with a variable presentation ranging from asymptomatic to nonspecific abdominal symptoms to perforation with frank peritonitis. Given its low incidence, jejunal diverticulitis can easily be overlooked in the working differential diagnosis; however, it is critical to maintain a high degree of clinical suspicion. In cases of complicated jejunal diverticulitis, early recognition and prompt surgical intervention are critical to improving patient outcomes.
